# Nanopore Assay Reveals Cell-Type-Dependent Gene Expression of Vesicular Stomatitis Indiana Virus and Differential Host Cell Response

**DOI:** 10.3390/pathogens10091196

**Published:** 2021-09-15

**Authors:** Balázs Kakuk, András Attila Kiss, Gábor Torma, Zsolt Csabai, István Prazsák, Máté Mizik, Klára Megyeri, Dóra Tombácz, Zsolt Boldogkői

**Affiliations:** 1 Department of Medical Biology, Faculty of Medicine, University of Szeged, 6720 Szeged, Hungary; kakuk.balazs@med.u-szeged.hu (B.K.); kiss.andras.attila@med.u-szeged.hu (A.A.K.); torma.gabor@med.u-szeged.hu (G.T.); csabai.zsolt@med.u-szeged.hu (Z.C.); prazsak.istvan@med.u-szeged.hu (I.P.); mizikmate@gmail.com (M.M.); tombacz.dora@med.u-szeged.hu (D.T.); 2 Department of Medical Microbiology and Immunobiology, Faculty of Medicine, University of Szeged, 6720 Szeged, Hungary; megyeri.klara@med.u-szeged.hu

**Keywords:** vesicular stomatitis virus, VSIV, transcriptome, nanopore sequencing, long-read sequencing

## Abstract

*Vesicular stomatitis Indiana virus* (VSIV) of genus *Vesiculovirus*, species *Indiana* *Vesiculovirus* (formerly as *Vesicular stomatitis virus*, VSV) causes a disease in livestock that is very similar to the foot and mouth disease, thereby an outbreak may lead to significant economic loss. Long-read sequencing (LRS) -based approaches already reveal a hidden complexity of the transcriptomes in several viruses. This technique has been utilized for the sequencing of the VSIV genome, but our study is the first for the application of this technique for the profiling of the VSIV transcriptome. Since LRS is able to sequence full-length RNA molecules, it thereby provides more accurate annotation of the transcriptomes than the traditional short-read sequencing methods. The objectives of this study were to assemble the complete transcriptome of using nanopore sequencing, to ascertain cell-type specificity and dynamics of viral gene expression, and to evaluate host gene expression changes induced by the viral infection. We carried out a time-course analysis of VSIV gene expression in human glioblastoma and primate fibroblast cell lines using a nanopore-based LRS approach and applied both amplified and direct cDNA sequencing (as well as cap-selection) for a fraction of samples. Our investigations revealed that, although the VSIV genome is simple, it generates a relatively complex transcriptomic architecture. In this study, we also demonstrated that VSIV transcripts vary in structure and exhibit differential gene expression patterns in the two examined cell types.

## 1. Introduction

Vesicular stomatitis Indiana virus (VSIV) is a negative single-stranded RNA virus belonging to the Rhabdoviridae family [1]. The virus causes a zoonotic disease, and the infection spreads between mammalian hosts via insect bites or direct contact [2]. Although the virus causes only mild symptoms in humans [3,4], including fever, myalgia, headache, vomiting [5], enlarged lymph nodes and conjunctivitis, it causes vesicular disease in animals including horses, cattle and, especially, pigs [6], which are the natural host of the virus [7]. The vesicular disease is very similar to foot and mouth disease, and thus can lead to losses in domestic livestock and therefore to significant economic loss. VSV infection used to be common among laboratory workers and animal handlers as well.

The VSIV genome is small (11,161 kb) [2], encoding five polypeptides: N, P, M, G, and L. The large L protein is an RNA-dependent RNA polymerase (RdRp), which is also responsible for capping and polyadenylation of VSIV mRNAs [8,9]. The nucleoprotein (N) [10] surrounds the RNA molecule. The phosphoprotein (P) is a catalytic cofactor for the L protein [11]. The matrix protein (M) performs many functions, including assembly, packaging, apoptosis, and blocking of host RNAs [12,13]. Glycoprotein (G) is responsible for the entry into the host cells [14,15]; it is required for the attachment of the virion to the cell surface Low-Density Lipoprotein (LDL) receptor [16], enabling the virus to enter the cell via receptor-mediated endocytosis [17]. The acidity of the endosome lumen causes a conformational change in the G protein, thereby activating it [18]. The fusion between the viral envelope and the endosomal membrane is then facilitated by the activated G protein, which leads to the release of the viral helical nucleocapsid into the cytoplasm of the host cell.

The (−) strand VSV RNA serves as a template for transcription of the five major mRNAs. The viral capsid contains small amounts of L-protein and phosphoproteins that can initiate viral RNA synthesis after the intrusion [19]. The transcription and replication of VSIV begins at the 3′ end of the genomic RNA. The viral RdRp functions in two modes [20]: in replication, it produces a full-length RNA molecule; whereas in transcription, the individual mRNA molecules are synthesized. The viral mRNA synthesis occurs in specialized liquid compartments, called viroplasms [21].

Free cytoplasmic ribosomes are involved in the translation of four proteins (N, P, M, and L), but the G protein is translated by another pathway that is controlled by endoplasmic reticulum-bounded ribosomes [22]. The newly synthesized proteins prepare a full-length complementary (+) strand RNA which serves as a template for the synthesis of the (−) strand RNA genome. This in turn is incorporated into the progeny virions. The newly synthesized G protein is glycosylated and oligomerized [23], then enters the secretory pathway, and is transported to the plasma membrane. When every other viral component gets transported, G protein initiates the assembly of virions, which can now egress from the host cell, being ready for the next infection [24].

Next-generation short-read sequencing (SRS) techniques can accurately characterize viral gene expression [25], but do not provide high-resolution details of the various transcript isoforms, and multigenic and overlapping transcripts. The emerging long-read sequencing (LRS) techniques can circumvent these limitations because they are able to read full-length transcripts [26,27]. The LRS techniques have been becoming increasingly popular in viral genome and transcriptome researches, and the studies based on these platforms report an unexpectedly large complexity of the viral transcriptomes [28,29,30,31]. An LRS approach has already been used for the sequencing of the VSIV genome [32], but our study was the first for the application of this technique for the profiling of VSIV transcriptome.

In this work, we investigated the structural and kinetic aspects of the polyadenylated fraction of the VSIV transcriptome in fibroblast and glial cell lines using the ONT MinION amplified and non-amplified cDNA sequencing techniques. Furthermore, we described the detected significant differences in the gene expression dynamics of two host cells.

## 2. Methods

The experimental system used in this study is shown in Supplementary Materials Figure S1. We used two biological replicates for the cell cultures as well as subsequent library preparation and sequencing.

### 2.1. Cells and Viral Infection

Strain Indiana of VSIV was propagated on African green monkey kidney fibroblast (Vero) and on human glioblastoma (T98G) cell lines (ECACC). Cells were grown in DMEM (Gibco/Thermo Fisher Scientific, Waltham, MA, USA), supplemented with 5% fetal bovine serum (Gibco/Thermo Fisher Scientific, Waltham, MA, USA) and 80 μg/mL gentamycin (Gibco/Thermo Fisher Scientific, Waltham, MA, USA) at 37 °C in the presence of 5% CO_2_. Both cell types were infected with a high multiplicity of infection (MOI = 5), and samples were taken at multiple time points (1 h, 6 h, 15 h, 24 h).

### 2.2. Isolation of RNA

Total RNAs were isolated using NucleoSpin^®^ RNA kit (Macherey-Nagel, Düren, Germany) according to the manufacturer’s recommendation. Samples were treated with Ambion^®^ TURBO DNA-free™ kit to eliminate residual DNA contamination. The concentration of RNA samples was determined using a Qubit^®^ 4.0 Fluorometer and the Qubit RNA BR Assay Kit (Life Technologies, Carlsbad, CA, USA). The poly(A)+ RNA fraction was isolated applying Oligotex mRNA Mini Kit (Qiagen, Hilden, Germany).

### 2.3. Oxford Nanopore MinION Sequencing

For the preparation of cDNA libraries, the polyA(+) RNA fraction was reverse transcribed using an oligo(d)T-containing primer [(VN)T20 (Bio Basic, Markham, ON, Canada)]. The RT reaction carried out using SuperScript IV enzyme (Life Technologies, Carlsbad, CA, USA), a strand-switching oligo [containing three O-methyl-guanine RNA bases (PCR_Sw_mod_3G; Bio Basic, Canada)] added to the sample. The cDNAs were amplified using LongAmp Taq 2× Master Mix (New England Biolabs, Ipswich, MA, USA) and Ligation Sequencing Kit Primer Mix according to the ONT Kit’s manual. End repair was made on the samples using the NEBNext End repair/dA-tailing Module (New England Biolabs). The “barcoding” was made by the specific barcode (ONT PCR Barcoding Kit 96; EXPPBC096), and ligated to the sample according to the 1D PCR barcoding genomic DNA (SQK-LSK109) protocol. Barcoded samples were amplified by PCR using LongAmp Taq 2× Master Mix. The PCR product was end-repaired, then it was followed by adapter ligation utilizing the sequencing adapters supplied in the kit and in the NEBNext Quick Ligation Module (New England Biolabs, Ipswich, MA, USA). The cDNA sample was purified between each step using Agencourt AMPure XP magnetic beads (Beckman Coulter). To avoid the analysis of potential false PCR products, non-amplified cDNA libraries were also prepared using the ONT’s Direct cDNA (dcDNA) Sequencing Kit (SQK-DCS109), according to the manufacturer’s recommendations, as described earlier [33]. The amplified libraries were run on MinION SpotOn Flow Cells (R9.4), while the dcDNA samples were loaded onto ONT Flongle Flow Cells.

### 2.4. Cap Selection Protocol

For capturing the 5′-cap structure, a specific adapter was ligated to the cDNAs using the Lexogen’s TeloPrime Full-Length cDNA Amplification Kit (25 °C, overnight). The samples were amplified by PCR using the Enzyme Mix and the Second-Strand Mix from the TeloPrime Kit. Detailed protocols can be found in our earlier publication (https://www.nature.com/articles/sdata2018119 (accessed on 5 July 2021)). The reactions were performed in a Veriti Thermal Cycler, and the samples purified on silica membranes (TeloPrime Kit) after the enzymatic reactions. The sequencing-ready libraries were loaded onto R9.4 SpotON Flow Cells.

### 2.5. Bioinformatic Analyses

Guppy software v3.3.3 (ONT) was used for base calling of the data from MinION sequencing. The raw reads were aligned to the Vesicular stomatitis Indiana virus reference genome (NCBI Nucleotide accession: NC_001560.1) using *minimap2* with the following options: -ax splice -Y -C5 -cs. The LoRTIA software (https://github.com/zsolt-balazs/LoRTIA (accessed on 5 July 2021)), which can filter out false products, was used to find TESs and TSSs and to annotate viral transcripts using default parameters for the ONT platform. We used additional criteria for the annotations to eliminate potentially spurious transcripts: only those features were accepted as true that were detected in least two amplified cDNA and in one dcDNA library. Coding capacity estimation was carried out with the Coding Capacity Assessment Tool (http://lilab.research.bcm.edu/index.php (accessed on 6 September 2021)).

The host cell’s gene abundance estimation was carried out with salmon [34] on the GCA_000409795.2 and GCA_000001405.28 genome assemblies for the vervet monkey fibroblast and Human glia cells, respectively. Transcript counts were summed per gene and then translated to the SYMBOL database, as gene SYMBOLS for most genes are shared between *H. sapiens* and *C. sabaeus*. In order to be able to assess the differences between the two host cell lines, those genes whose gene symbol was not found in the other cell line were filtered out, i.e., only the intersection of gene SYMBOLs were used in the downstream analysis. On average, ~73% of all transcript counts from each sample could be assigned into gene SYMBOLS. ImpulseDE2 [35] (utilizing DESeq2 [36]) was used to identify genes that showed differential kinetic profiles between the two host cell lines and within each cell line (Differentially Expressed Genes, DEGs). Clusterprofiler [37] was used to assess which KEGG pathways were significantly different, based on the DEGs. Complexheatmap [38], Gviz [39], and the packages of the tidyverse [40] were used for data analysis and visualization in R [41].

## 3. Results

### 3.1. Time-Course Long-Read Sequencing of the VSIV Transcriptome

Our investigations revealed that the simple VSIV genome encodes a relatively complex transcriptomic architecture, which differs in the two investigated cell lines with regard of both the structure and the kinetics of transcripts. Two technical replicates were used from the amplified cDNA-Seq samples at each time point (1, 6, 15, 24 hpi) in both cell lines. Direct cDNA sequencing (dcDNA-Seq) was used to confirm transcript identity. The read length distribution is illustrated in Supplementary Materials Figure S2. The detailed sequencing statistics of VSIV and host cells are shown in Supplementary Materials Table S1.

The obtained reads were analyzed using LoRTIA for the identification of transcription start sites (TSSs) and transcription end sites (TESs). Before filtering, 166 TSSs and 76 TESs were annotated, but as these were not consistently detected throughout the samples, many of them were filtered out. The stringent filtering criteria that we used to accept these transcription features (TES and TSS) lead to the identification of nine high-confidence novel TSS; however, all non-canonic TESs were filtered out. Thus, it should be noted that some of these low-confidence TESs (and perhaps TSSs as well) may exist, but further experiments are needed to validate them. The low-confidence, putative TSSs and TESs are listed in Supplementary Materials Table S2/TSS and Table S2/TES, respectively.

### 3.2. Novel Transcripts of VSIV

**Figure 1:**
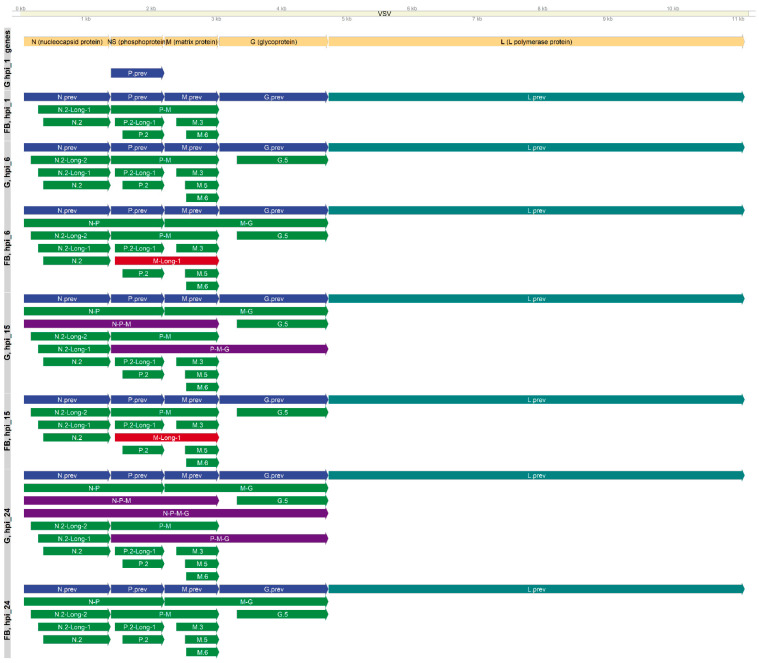
Kinetic transcriptome profiles of VSIV. The set of annotated transcripts in each time point and in each cell line is shown. Yellow arrows indicate the VSIV genes; blue arrows illustrate the previously described canonical transcripts; green arrows illustrate transcripts that are identical in both cell types; indigo arrows indicate transcripts found only in glial cells; and dark red arrows indicate transcripts found only in fibroblast cells. The L gene was detected only in fragments; that is, no read that spans the entire transcript was found. This is indicated with a teal color.

The transcripts were annotated by finding reads that mapped from start to end to a high-confidence TSS and a TES using the LoRTIA program. The transcript annotation data were obtained, including read counts for the transcripts in each sample, and their estimated coding capacity is presented along with other inferred information regarding the transcripts (source, category, gene) in Supplementary Materials Table S2/LoRTIA. With this approach we identified a total of 16 novel transcripts (in both cell lines) expressed from the VSIV genome that met our filtering criteria (Figure 1). Moreover, we found differences in the sets of VSIV transcripts that are produced in the two host cells during the viral infection.

Of the herein identified novel transcripts, nine were nested RNAs. These contain 5′-truncated in-frame ORFs that are embedded into the longer canonical ORFs; thus they are the products of putative nested genes and might encode N-terminally truncated polypeptides. The ORFs were predicted in silico and are listed in Supplementary Materials Table S2/ORFs.

Three embedded transcripts were found to be expressed from the host M genes. This is in line with previous studies [42] that described two N-terminally truncated proteins from this gene, translated independently from the M1 protein via alternative downstream start codons and by a leaky ribosomal scanning mechanism (described also in the phosphoprotein gene of rabies virus [43]). The embedded M3 transcript corresponds to one of these truncated proteins (M3). The two additional embedded transcripts also contained predicted in-frame ORFs. However, only M3 surpassed the threshold (50% coding probability estimated via the CPAT tool) to be regarded as coding (63%); these other two transcripts (M5 and M6) had a low coding capacity estimation (25–30% probability of coding), probably because these transcripts get translated rarely (Supplementary Materials Table S2/CPAT results).

A single embedded transcript from the G gene and three from the N gene (two being longer TSS variants of the same nested gene) were detected, and all estimated to be coding. In addition, two embedded transcripts were identified from the P gene, and although they carry a short in-frame ORF (99 AA), their coding probability was estimated to be very low. While the longer variant contains the C and C’ ORF [44], it is unlikely that this transcript would be translated into the C’ protein, as the transcript is co-terminal with the canonic transcript (and the shorter embedded). It is more likely that if this transcript encodes a protein after all (despite the low coding probability) that would be an N-terminally truncated version of the P protein and neither C nor C’. Another plausible explanation is that these transcripts (along with the two short, truncated transcripts of the M gene) are non-coding transcripts and may be involved in transcript regulation.

Bi- and multicistronic viral mRNAs were also detected in both host cells. In fibroblast cells, only bicistronic mRNAs were found; however, in the glial cells, we detected five multigenic transcripts as well. Three different bicistronic mRNAs were found in both cell types: N-P, P-M, and M-G. Most likely, the first ORF was translated from these transcripts, however it is also possible that the virus induces an alteration in the host translation machinery and thus more ribosomes fail to initiate at the first AUG, leading to the leaky ribosomal scanning mechanism and the translation of downstream ORFs.

### 3.3. Kinetic Analysis of VSIV Transcripts

**Figure 2:**
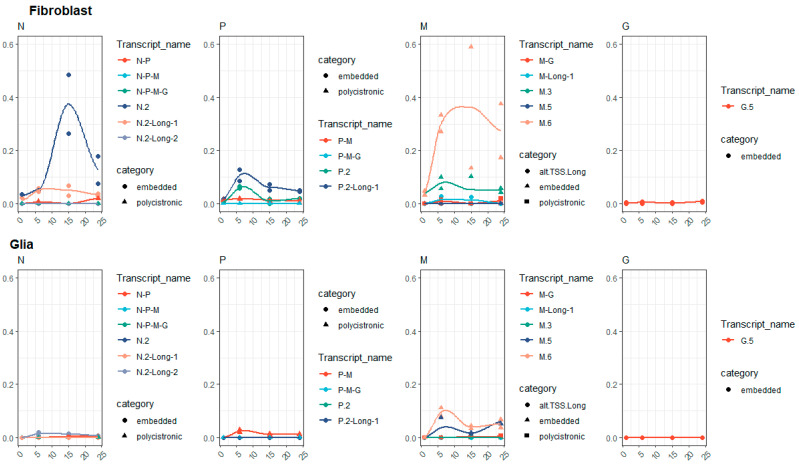
Relative transcript category expression. This chart illustrates the proportion of different transcript categories (embedded, polygenic, long alternative TSS isoform) compared to the canonic transcripts. The values were calculated as the sum of the expression values of the transcripts in each gene and in each sample divided by the expression values of the canonic transcript in the respective gene and sample.

The kinetic analysis of the annotated VSIV transcripts carried out in both cell types at four sampling time points revealed a differing structural and temporal expression pattern of the VSIV genes in the two cell lines, including the 5′-truncated RNAs. There is a remarkable dissimilarity between the proportions of 5′-truncated and canonic transcripts of the genes at each time point in the two cell types (Figure 2). Generally, fibroblast cells produced a higher percentage of embedded transcripts. Except for the L (where no high-confidence isoform was detected) and G genes, each expressed relatively high proportions of embedded genes. Interestingly, the proportion of these truncated transcripts followed a similar pattern: very low percentage at the start of the infection, a peak at either 6 or 15 hpi; and decrease at 24 hpi. These can be viewed as isoform-switching events. The highest proportion of 5′-truncated transcripts was observed in the case of M and N genes: here 60–75% percent of the gene’s expression was composed of these truncated transcripts (although, in the case of the embedded M gene, Fibroblast hpi 15 sample there was a larger deviation.

Even moreso, this pattern was seen in the case of the M gene in Glia cells as well, only here the proportions were lower but the shape of curve is clearly similar. This suggests that the expression of these truncated transcripts is regulated differently from their host (canonical) transcripts and is also differentially in the two cell lines. In the case of the P gene, the polygenic transcripts showed a similar pattern but were lower in proportion; the proportions of the polycistronic RNAs were generally low. In both cell lines, the highest values were detected in the P gene, 6 hpi samples: 1.8% in glial cells and 5.5% in fibroblast cells. The proportions fluctuated below these values without a clear trend their expression profile.

### 3.4. Viral Gene-Level Expression Kinetics

**Figure 3:**
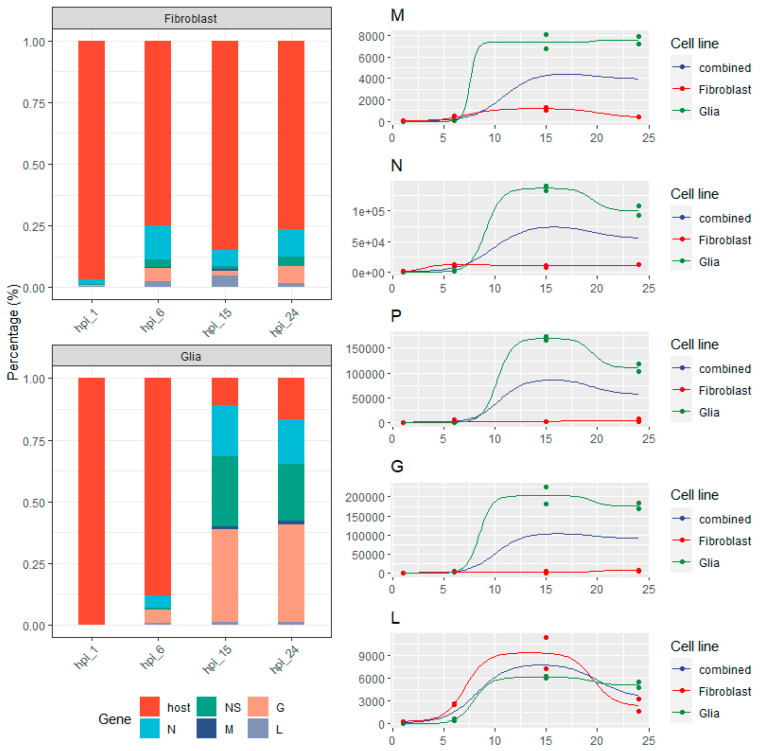
Gene-level expression kinetics. The left panel (stacked bar chart) shows the proportion of reads in each library as estimated by *salmon*; the filling colors show the origin of the reads (host cell or one of the viral genes). The right panel shows the expression trajectory of each viral gene across the time points and the impulse model fitted on the data. Colors represent the two cell lines and the combined model from both cell lines.

Viral gene expression values were estimated with *salmon*, and the resulting count matrices were evaluated with ImpulseDE (which uses DESeq2′s normalization approach), to analyze each gene’s expression level as the function of time (gene expression difference between samples). The right panel in Figure 3 shows the normalized read counts for each viral gene in the samples, whereas the left panel shows the relative abundances (ratio of all reads) of the viral genes and the sum of the host reads.

The N, P, M and G genes showed a somewhat similar expression pattern in the Glia cells: only a very low amount of viral reads were obtained in the 1 h post infection (hpi) samples (a total 30 and 31 reads could be pseudomapped to the viral transcriptome in the two replicates, respectively); viral transcription kick-started in the hpi 6 samples, which was followed by a considerable increase and a peak at hpi 15; and, finally, expression decreased at hpi 24 in the case of P and N but decreased only slightly in the case of G or remained more or less the same in the case of the L and M genes. The shape of the gene expression curve in the case of fibroblast cells was similar except for the P and G genes, where it fluctuated in the 6, 15 and 24 hpi samples. The gene expression levels showed a striking difference, however: in glia cells the proportion of the viral reads elevated from only 11% in the hpi 6 samples, which is comparable to 21% in the fibroblast cells, to 89% in the hpi 15 samples and decreased only slightly in the hpi 24 samples (83%). This phenomenon may be due to the VSIV M protein, which blocks the escape of host mRNAs from the nucleus by blocking the nucleopores and preventing host RNAs from entering the cytoplasm [45,46]. In contrast, fibroblast cells followed a different trend. The number of viral mRNA at 1 hpi was 2.5% of all obtained reads. At 6 hpi, this was increased to 20%, but at the following timepoints it did not increase further. It seems that the Vero cells were able to form some kind of a balance with the virus and didn’t allow it to effectively inhibit their own gene expression, at least not until 24 hpi. These results show that there is a significant difference in viral gene expression levels and virus: to: host expression ratios between the two cell lines. One exception is the viral L mRNA, as its abundance levels showed no difference between the two cell lines; in both the glia and fibroblast cells, the L gene showed low expression values but in the fibroblast cells this accounted for a larger proportion of reads. The proteins formed from VSIV-N and VSIV-P mRNAs are cofactors for the virus’s RdRp, and they are involved in its regulation as well [19]. The elevated levels of these transcripts in glial cells might cause the upregulation of the RdRp, and hence might lead to a global increase of viral gene expression. Further experiments are needed to investigate this possibility.

### 3.5. Host Gene Expression

The gene expression count matrices as produced by salmon and translated to gene symbols (only shared symbols between *H. sapiens* and *C. sabaeus* were kept) were analyzed using ImpulseDE2, first in case-control mode. In this test, genes with a low p-value exhibited different kinetic profiles between the two cell lines, but not necessarily a difference between the initial (hpi 1) and subsequent time-points within the cell line. This showed that out of the 16,133 shared genes, 1370 were significantly differentially expressed (DEG) between fibroblast and glial cells under a FDR-corrected *p*-value cutoff of 0.01. This DEG list was supplied as an input for Clusterprofiler to identify KEGG pathways that are differentially expressed by analyzing the number of DEGs that are involved in the pathways. As a result, 35 pathways were found (Supplementary Materials Figure S3). Many of these are associated with viral diseases (i.e., Coronavirus, Influenza and Epstein-Barr infections), but we found several that are associated with more general cell function, i.e., carbon metabolism and ribosome. This is because many genes with widespread functions were differentially expressed between the two host cells. The host translation machinery is indeed affected by the viral infection, but its extent is apparently not the same between the two cell lines. It must be noted that Vero cells have a genetic defect in interferon production [47], and the fibroblast cell line (T98G) is hyperpentaploid. This may also explain some differences in their transcriptomic profile.

We also carried out an analysis separately for the two cell lines, which tests whether the gene expression deviates from a constant model (case-only mode). This showed that gene expression profiles changed significantly in 460 genes in glial cells, and in 176 genes in fibroblast cells. Overall, glia cells showed significantly more DEGs as a function of time. Seventy-one genes overlapped in this comparison; these are the genes whose expression seem to be affected the most by the viral infection in both cell lines. There were about three times more DEGs in the fibroblast vs. glia comparison than in the within-sample comparisons (Supplementary Materials Figure S4). The list of DEGs for each comparison is provided in Supplementary Materials. The DEGs in each cell line (case-only analyses) were clustered together according to their expression pattern into five clusters. The z-score normalized gene expression values in the two cell lines for these genes are shown in Supplementary Materials Figure S5/A-B (heatmap), and in Supplementary Materials Figure S6 (scatterplot). Supplementary Materials Figure S7 shows the z-score normalized gene expression for the viral genes. In fibroblast cells, cluster_3 showed a similar mean expression trajectory to that of the N gene and clusters 4 and 5 to that of M and L genes, while in glia cells, the expression pattern described above was similar to that of the host genes only in cluster_4, but came earlier in cluster_3 and with a delay in cluster_5. Overall, these results show that the effect of infection on host gene expression is completely different in the two cell types.

## 4. Discussion

In this work, several novel TSSs and associated transcripts of VSIV were identified using amplified and non-amplified cDNA nanopore sequencing and by a bioinformatic method that detected the entire length of reads that span from a TSS to the TES. We analyzed the kinetics of the transcripts in two cell lines during the viral infection. Bi- and polycistronic mRNAs, long TSS variants, and novel 5′-truncated mRNAs that are embedded in the longer canonical genes were identified. We detected transcripts for the M3 protein, but not for the M2, and in addition detected two more truncated transcripts termed M.5 and M.6 that carry even shorter co-terminal ORFs. We found that the VSIV genome expresses a different set of transcripts in the two cell types and, moreover, the relative abundance of the novel transcripts compared to the canonic transcript of the respective gene varies as a function of time and between the cell lines.

Most of the polygenic mRNAs were expressed in low abundance compared to the canonic transcripts; however, in some cases, mainly in the fibroblast cells, the newly identified 5′-truncated mRNAs were expressed in very high proportions. This was the most apparent in the case of the M gene. Regarding this gene, the truncated M.6 and M.3 transcripts in Fibroblast cells showed an expression of 10–50% of that of the canonic transcript (M), while in Glia cells the M.6, and M.5 transcript’s expression was about 5–10% and 1–5% of the expression of that of the canonic M transcript, respectively. Interestingly however, though all these transcripts contained in-frame ORFs, the coding probability for the M.5 and M.6 transcripts were estimated to be only 25–30%. This is rather low, but not unreasonable), thus even though these transcripts are expressed in relatively high amounts (compared to the canonic transcript) they may not encode proteins and have only regulatory functions. The embedded M3 and M2 proteins, similarly to the canonic M1 protein, were shown to induce cytotoxicity, cell rounding, and eventual cell death in BHK cells, although most likely M2 and M3 do not participate in the budding of VSV particles [13]. In our results, the fibroblast cells, where the truncated M mRNAs (including the M3) were expressed much higher in relative abundance, were far more resistant to the viral infection. Whether or not this is a mere correlation without any functionality can be experimentally verified. We also identified a transcript that carried the ORF for the C’ protein (P.2-Long-1), but their coding probability is very low (~11%). And because the transcripts and the predicted ORFs they carry are co-terminal with the canonic transcript and ORF, it is more likely that if they are translated after all, their product would be an N-terminally truncated version of the P-gene and not the C’, whose ORF is out of frame. The relative abundance was low in the fibroblast cells (around 5–10%) and even lower in the Glial cells.

Host gene expression changes were also evaluated. We found a significant difference in the effectiveness of viral infection between the two cell types. Glia cells are much more sensitive to infection, while fibroblast cells are more resistant. We detected 1370 differentially expressed genes and 35 differentially expressed KEGG pathways between the cell lines.

Using ImpulseDE2 in case-only mode independently for the two cell lines, about 2.5 times more genes were found in the glia cells, whose expression trajectory was significantly affected by the viral infection (452 in glia cells, and 172 in Vero cells). From these, 137 were found to also be involved (significantly changed) during rabies infection in mouse brain cells [48]. This suggests that these genes are likely affected in other cell lines and host species, and thus should be the subjects of further investigation regarding the pathogenesis of vesicular disease in livestock.

Viral gene-level expression values as a function of time were also determined and compared between the cell lines. Although the expression curves were similar in many cases, the glial cells showed significantly higher expression levels (also proportions, compared to the host gene expressions). Interestingly, their curves were similar to what was observed in the proportions of embedded and canonic transcripts of each gene. Besides the potential for the 5′-truncated transcripts to encode functional proteins, they may play a role in regulating gene expression. Indeed, it is possible that the isoform switching events, i.e., the change in the proportions of the truncated and canonic transcripts in the genes, contribute to the apparent resistance of the fibroblast cells against the viral infection, although this needs to be tested experimentally.

## Data Availability

Raw datasets are available in European Nucleotide Archive: PRJEB46127 (https://www.ebi.ac.uk/ena/browser/view/PRJEB4612) (accessed on 5 September 2021). The LoRTIA pipeline is available at GitHub: https://github.com/zsolt-balazs/LoRTIA (accessed on 5 September 2021).

## Supplementary Materials

The following are available online at https://www.mdpi.com/article/10.3390/pathogens10091196/s1, Additional File S1: Supplementary Figures S1–S7; Additional File S2: Supplementary Table S1; Additional File S3.
