# Supplementary material for: Nanopore Assay Reveals Cell-Type-Dependent Gene Expression of Vesicular Stomatitis Indiana Virus and Differential Host Cell Response
Source: Pathogens. 2021 Sep 15;10(9):1196. doi: 10.3390/pathogens10091196 (PMC8468008; doi:10.3390/pathogens10091196)
Supplement: Supplementary file 1 [file pathogens-10-01196-s001.zip › Additional-File-1-Figures.pdf]

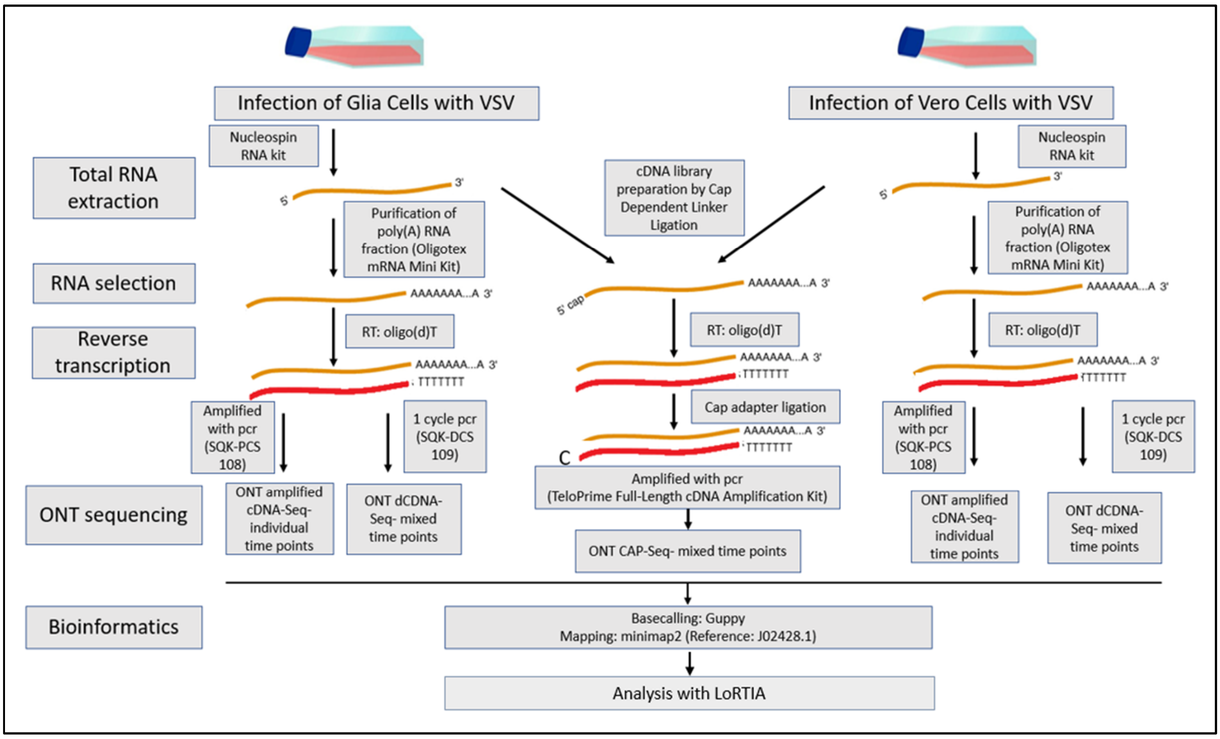

Additional File 1, Figure S1 ***The flowchart diagram shows the MinION (ONT) sequencing methodologies and the bioinformatic analysis that we use.***

Sequencing libraries were generated by three different methods (amplified cDNA (for individual time points), dcDNA (for mixed time points), and dcDNA by Cap-selection (for mixed time points)). The basecalling was done with the Guppy program. The resulting reads were mapped with minimap2. Further analyzes were performed using the Lortia program.

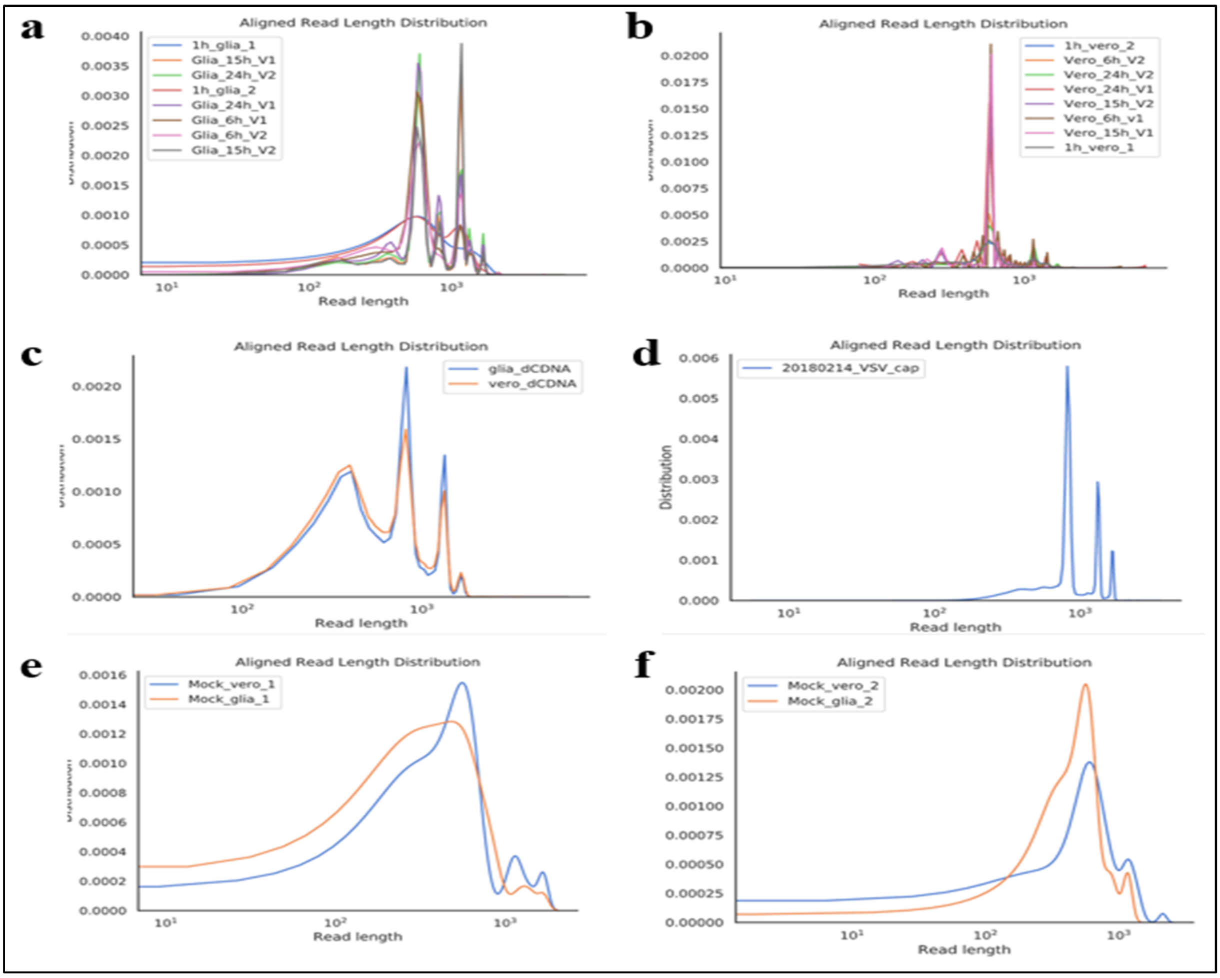

Additional File 1, Figure S2. Aligned read length density distribution in the two cell lines, obtained by ONT MinION sequencing. Amplified cDNA sequencing from glial (a) and fibroblast (b) cell types at different time points. Direct cDNA sequencing from glial and fibroblast cell types (c). cDNA sequencing of a mixture of samples obtained by Cap selection (d). cDNA sequencing of a mix of Mock 1 and 2 (e, f).

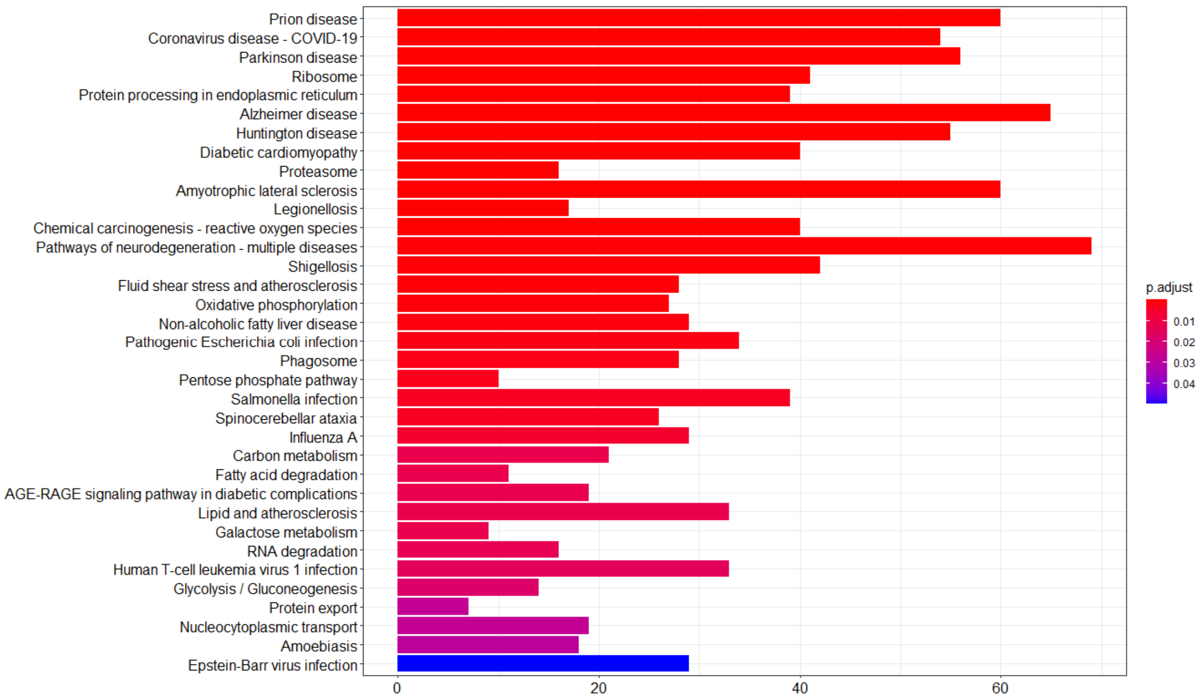

Additional File 1, Figure S3.

Differentially expressed KEGG pathways between the two cell lines. X-axis showa the number of differentially expressed genes (DEGs) in each pathway, while the colors of the bars correspond to the adjusted *p*-values of the test results.

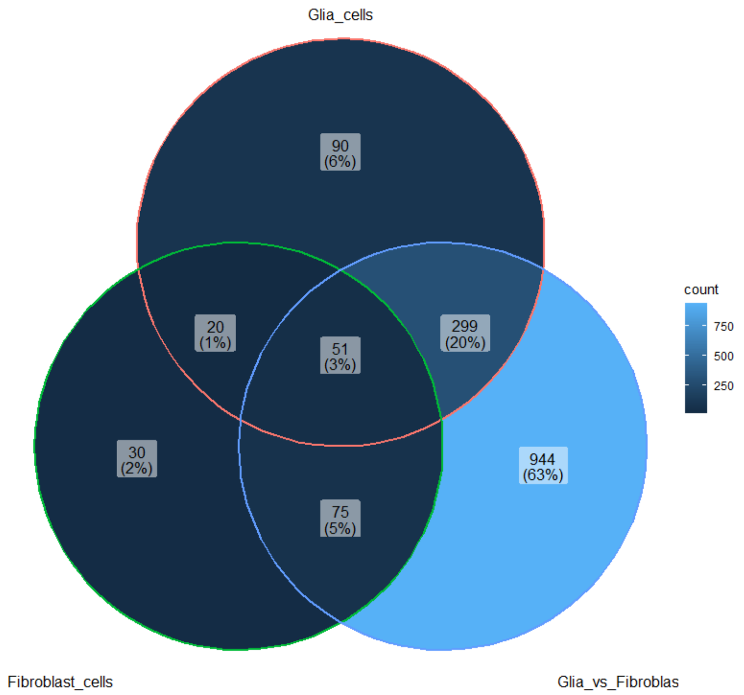

Additional File 1, Figure S4.

The number of differentially expressed genes (DEGs) in each comparison.

A.)

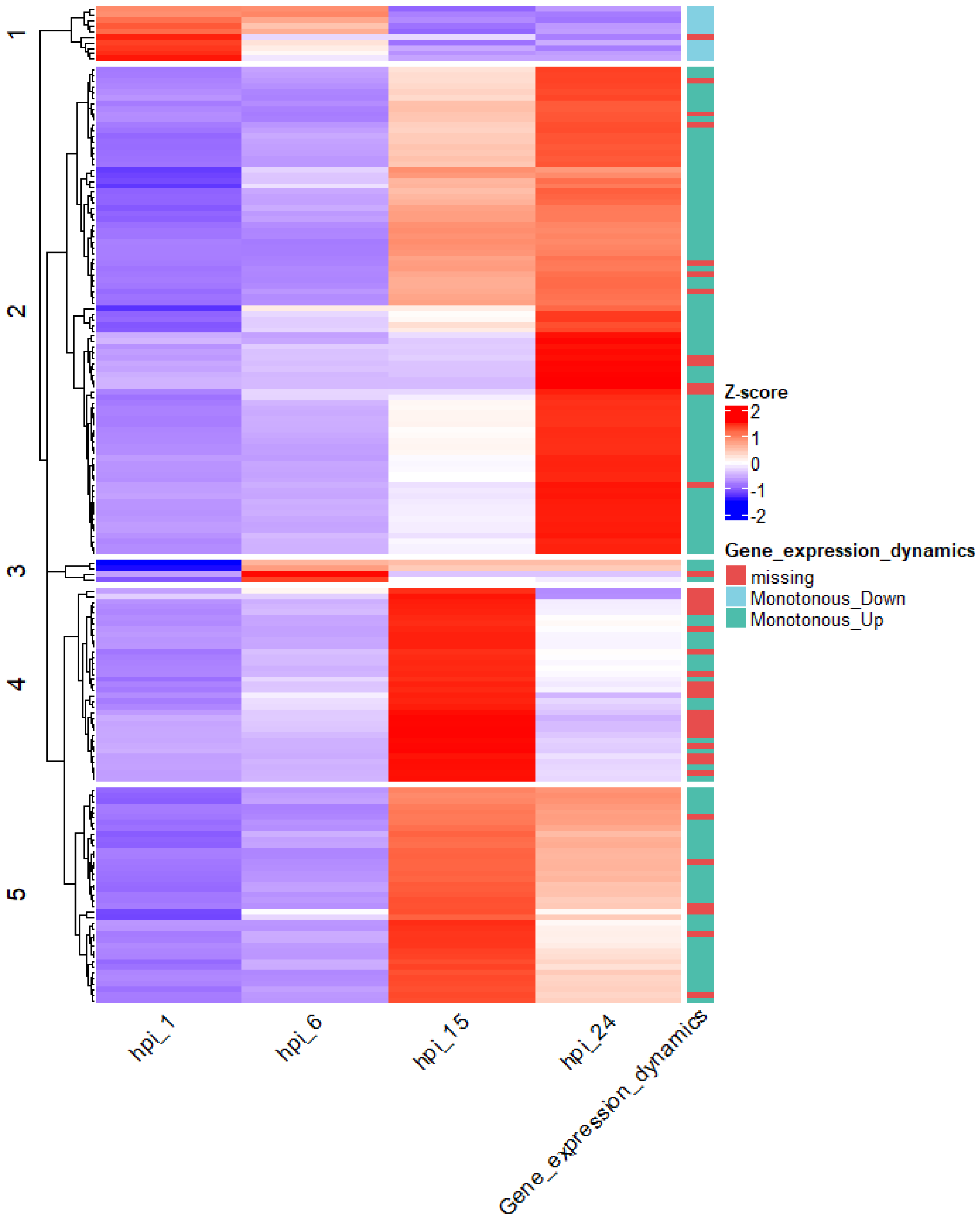
1
2
3
4
5
hpi_1
hpi_6
hpi_15
hpi_24
Gene_expression_dynamics
Z-score
2
1
0
-1
-2
Gene_expression_dynamics
missing
Monotonous_Down
Monotonous_Up

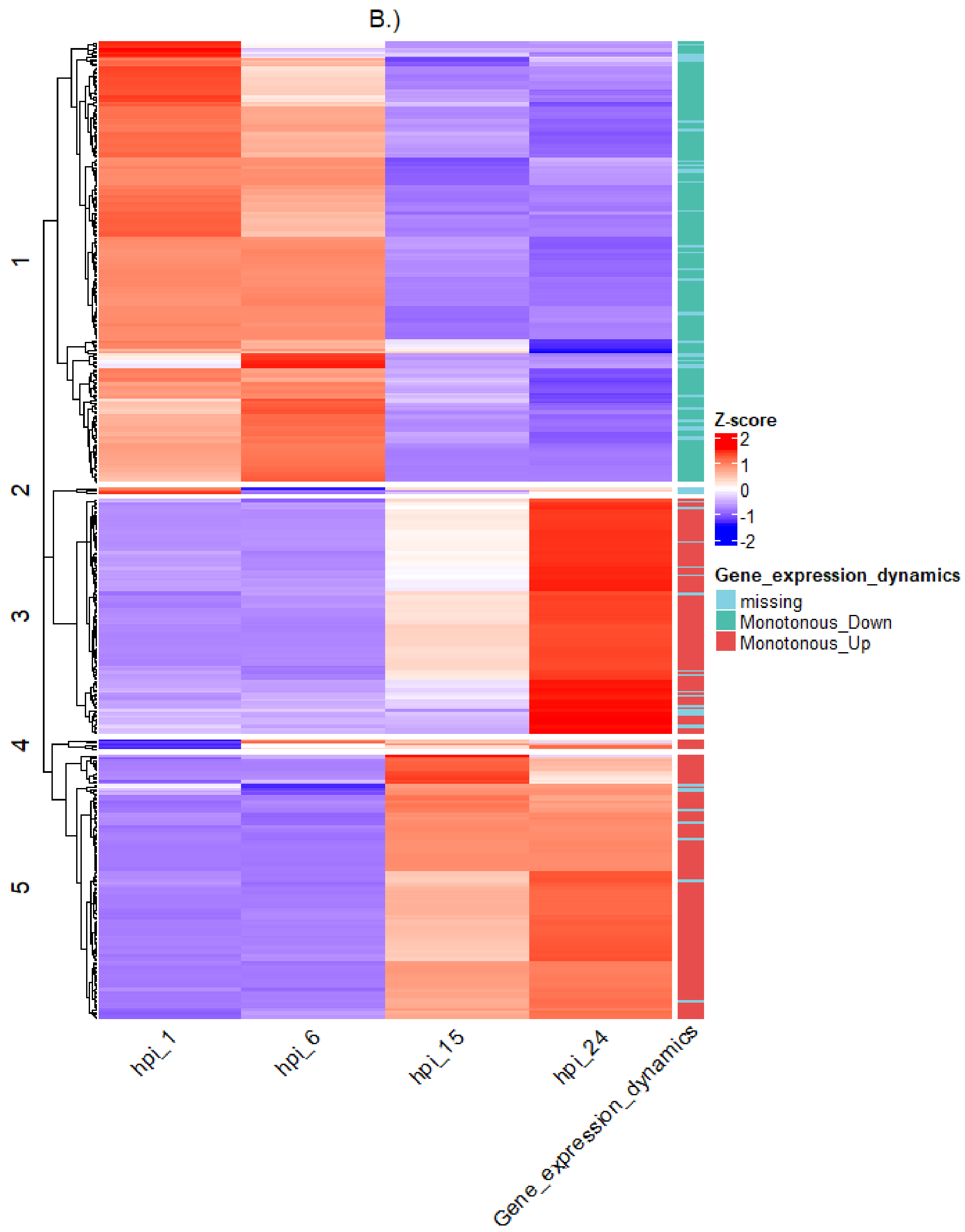

Additional File 1, Figure S5/A-B.

Heatmap of the z-score normalized gene expression kinetics in the two cell lines: **A:** Fibroblast; **B:** Glia. The annotation column on the right (gene expression dynamics) shows whether ImpulseDE2 characterized the given genes expression as either monotonously decreasing or increasing.

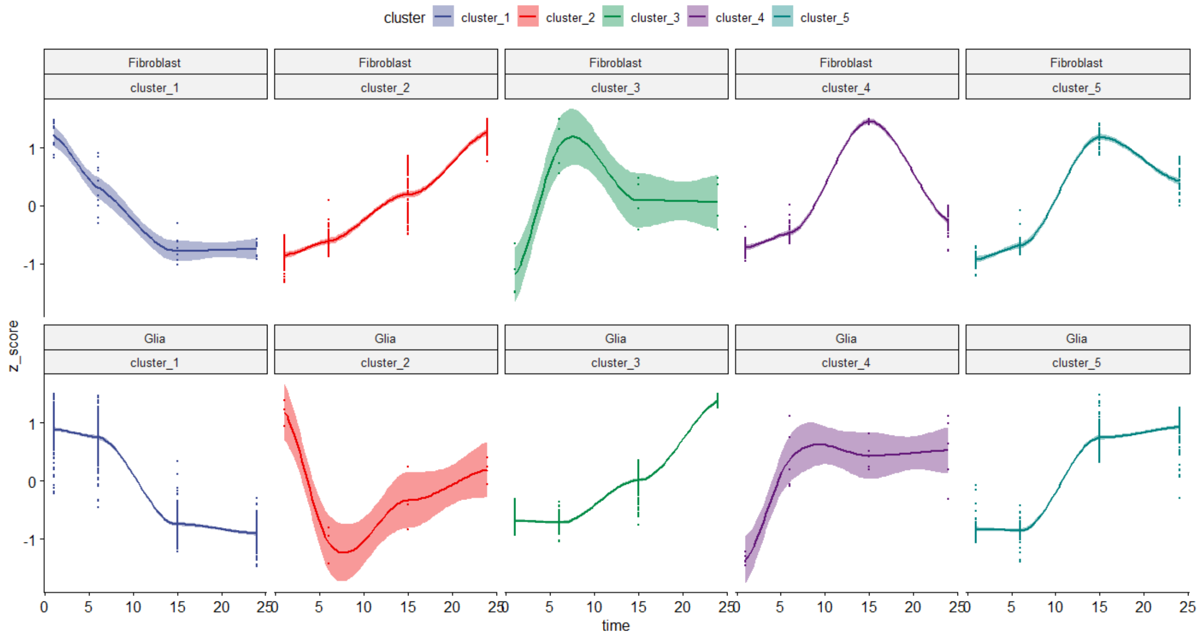

Additional File 1, Figure S6.

Z-score normalized host gene-expression values in each gene cluster in the two cell lines. The genes were clustered with k-means clustering. Shaded areas represent 95% confidence intervals.

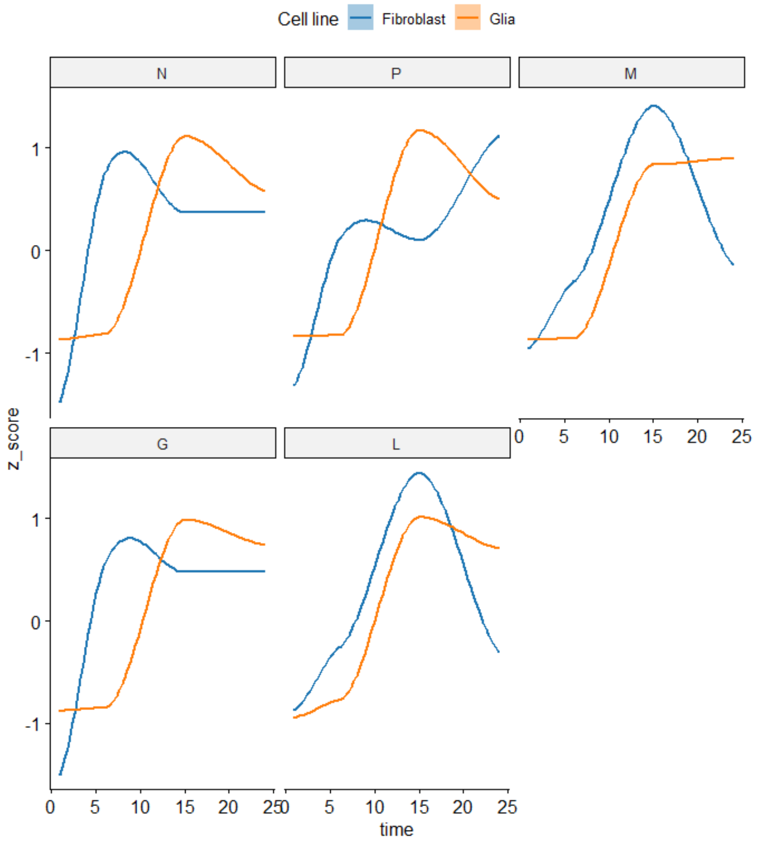

Additional File 1, Figure S7.

Z-score normalized viral gene-expression values in the two cell lines.
