# Supplementary material for: Nanopore Assay Reveals Cell-Type-Dependent Gene Expression of Vesicular Stomatitis Indiana Virus and Differential Host Cell Response
Source: Pathogens. 2021 Sep 15;10(9):1196. doi: 10.3390/pathogens10091196 (PMC8468008; doi:10.3390/pathogens10091196)
Supplement: Supplementary file 1 [file pathogens-10-01196-s001.zip › Additional-File-2-Table-S1.pdf]

Sequencing techniques	Host cell reads	IVV reads	Average length of viral RNAs in bp	Average coverage of viral reads
MinION (ONT)_VSV_cap_mix	6,302,618	559,408	1,004.27	50,335.6
MinION (ONT) Glia_dcDNA_mix	29,888	53,305	775.42	3,703.39
MinION (ONT) Fibroblast_dcDNA_mix	80,344	32,360	763.40	22,13.39
MinION (ONT) Glia_amplified_cDNA	3,114,992	54,5547	781.81	4,251.85
MinION (ONT) Fibroblast_ amplified_cDNA	5,872,662	494,827	709.90	3,067.90

Additional file 2: Table S1. Reads and read counts obtained by using different sequencing techniques
